# Gut microbiota in sarcopenia and heart failure

**DOI:** 10.20517/jca.2022.07

**Published:** 2022-07-05

**Authors:** Chia-Feng Liu, W. H. Wilson Tang

**Affiliations:** 1 Center for Microbiome and Human Health, Department of Cardiovascular and Metabolic Sciences, Lerner Research Institute, Cleveland, OH 44195, USA; 2 Department of Cardiovascular Medicine, Heart, Vascular and Thoracic Institute, Cleveland Clinic, Cleveland, OH 44195, USA

**Keywords:** Gut microbiota, sarcopenia, heart failure, aging

## Abstract

Sarcopenia is common in aging and in patients with heart failure (HF) who may experience worse outcomes. Patients with muscle wasting are more likely to experience falls and can have serious complications when undergoing cardiac procedures. While intensive nutritional support and exercise rehabilitation can help reverse some of these changes, they are often under-prescribed in a timely manner, and we have limited insights into who would benefit. Mechanistic links between gut microbial metabolites (GMM) have been identified and may contribute to adverse clinical outcomes in patients with cardio-renal diseases and aging. This review will examine the emerging evidence for the influence of the gut microbiome-derived metabolites and notable signaling pathways involved in both sarcopenia and HF, especially those linked to dietary intake and mitochondrial metabolism. This provides a unique opportunity to gain mechanistic and clinical insights into developing novel therapeutic strategies that target these GMM pathways or through tailored nutritional modulation to prevent progressive muscle wasting in elderly patients with heart failure.

## INTRODUCTION

Heart failure (HF), a complex clinical syndrome that results from various types of heart disease, is the top killer of the human population, taking an estimated 17.9 million lives each year^[1]^. It is estimated that 6 million people in the US have the HF currently and the prevalence of HF is projected to increase by 46% from 2012 to 2030, affecting more than 8 million people^[2]^. A major risk factor for heart disease leading to HF is age^[3]^. The incidence of HF increases dramatically with each decade of life. For instance, HF incidence is between 6.0 to 7.9 per 1000 person-years after the index age of 45 years and increase to 21.1 per 1000 person-years after 65 years of age^[2]^. The prevalence of HF among US adults in middle age (40-59 years of age) is 1.6 % and 1.1% in man and women respectively but it is increased to 9.5 % and 11% in men and women greater than 80 years of age^[2]^. The lifetime risk for developing HF is around 38% at 90 years of age. It is clear that increasing prevalence of HF is associated with aging population. Furthermore, HF is the leading cause of hospitalization in older adults and more than 90% of HF deaths occur in patients older than 65 years^[4,5]^. The most common comorbidity among older patients with HF is sarcopenia.

The term sarcopenia, was initially used by Rosenberg in 1989 as an age-dependent condition of the loss of muscle mass and function^[6]^. The current updated definition of sarcopenia is now considered as a muscle disease rooted in decrease of muscle strength, reduction of muscle quantity and quality (muscle wasting) and decline of physical performance according to the European Working Group on Sarcopenia in Older People^[7]^. Sarcopenia is common among older adults with a prevalence between 10% and 40% but can also occur earlier in life when associated with systemic disease linked to inflammatory processes, e.g., malignancy or organ failure^[7]^. It was shown that the prevalence of sarcopenia in patients with HF was nearly 20% higher than the control subjects of the same age^[8–11]^. Mechanistically, HF and sarcopenia may share common determinants such as inflammation, hormonal alterations, malnutrition, oxidative stress and mitochondria dysfunction^[12,13]^.

Recent interests have focused on gut microbiota in human diseases and healthy aging. The human gut microbiota consists of a huge number of microorganisms. Among them, anaerobic *Bacteroidetes* and *Firmicutes* contribute > 90% of the total bacterial species in healthy guts. Perturbation of the gut microbiota or dysbiosis contributes to various diseases, including cardiovascular diseases (CVD), muscle wasting, and aging^[14]^. Understanding the interplay between gut microbiota and heart/skeletal muscle may help us to better identify vulnerability and investigate targeted interventions.

This review focuses on recent advances in our understanding of the interplay between the gut microbiota and CVD, emphasizing the role of cardiovascular aging and sarcopenia in HF. We examine the emerging evidence for the influence of the gut microbiome-derived metabolites in the development and progression of sarcopenia and HF. We also discuss several notable signal molecules and signaling pathways involved in both sarcopenia and HF and the contribution of gut microbiome during these processes.

## MUSCLE FUNCTIONAL DECLINE AND WASTING IN HEART FAILURE

In patients with HF, muscle wasting (or “sarcopenia”) is one of the major causes of exercise intolerance, ventilatory inefficiency, chronotropic incompetence, and insulin resistance^[5,7–11]^. These conditions may induce other clinical disorders and eventually affect individuals’ quality of life. Although this phenomenon has been well recognized by clinicians over centuries, formal definitions of sarcopenia continue to evolve and are not standardized. Nevertheless, the contemporary focus on muscle wasting as a therapeutic target in HF began almost three decades ago when Coats *et al*. proposed the “muscle hypothesis” that cardiac impairment in HF may promote catabolism and reduce anabolic factors that can lead to skeletal muscle abnormality^[15]^. This hypothesis provided the explanation that HF patients develop progressive exercise intolerance above and beyond impairment in their cardiac reserve and may explain the increased sympathetic activation, vasoconstriction, endothelial dysfunction, and ultimately worsening of left ventricular contractile function. Based on this hypothesis, the imbalance of catabolic and anabolic processes in skeletal muscle metabolism is a key trigger of muscle wasting. Differential expression of the signaling molecules and pathways controlling muscle growth, structure, and myocyte homeostasis have been described in HF and the elderly. This review focus on skeletal muscle atrophy/wasting in aging and HF. Cardiac muscle atrophy, which is usually induced by cancer treatment, will not be discussed here. We would like to direct readers to these review articles for further reading^[16,17]^.

### Signal molecules involved in skeletal muscle metabolism

**Figure 1.**
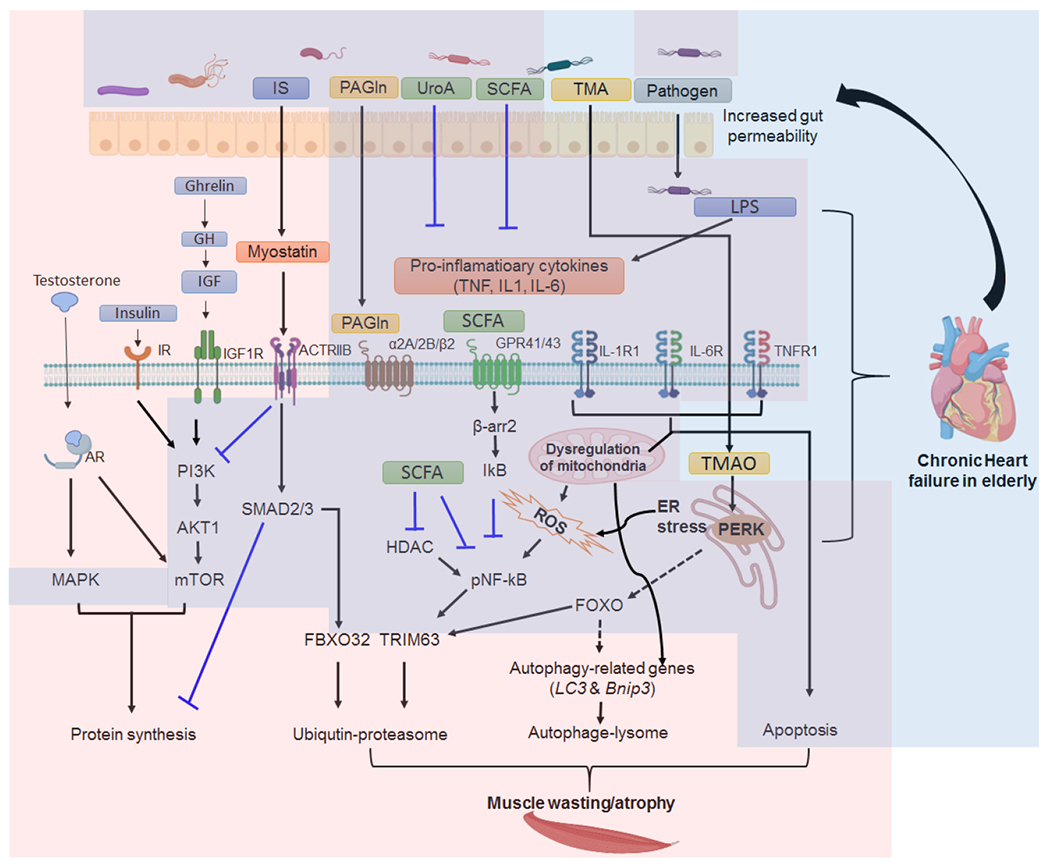
Gut microbiota dysbiosis-associated signaling pathways in muscle wasting and heart failure. Signaling pathways involved in skeletal muscle protein synthesis include androgen/testosterone, insulin and growth factors related pathways. Testosterone binds to its androgen receptor (AR) then together enter to nucleus to activate its downstream genes associated with muscle protein synthesis. It can also directly or indirectly influence mitogen-activated protein kinases (MAPK) and mammalian target of rapamycin (mTOR) pathways to control protein synthesis. IGF-1 and insulin elicit their protein sythesis function by binding to their receptors on cell membrane. Signaling pathways invovled in skeletal muscle wasting/atrophy include myostein-mediated transformation growth factor β signaling pathway, inflammation-assocaited signaling pathways. The ceullar mechamis asscoaited with skeletal muscle wasting/atrophy include the UPS proteiolsys pathway, autophage-lysome (ALP) and apopotsis. Dysregulation of mitochondria can trigger UPS, ALP and apoptosis leading muscle wasting/astrophy. Some gut microbial metabolites (GMM) and pathogens have anti-inflammation effect, such Short-chain fatty acids (SCFAs) and Urolithin A (UroA) and thus prevent muscle degredation. A variety of GMM and pathogens can trigger myostatin-mediated SMAD2/3 signaling pathway and inflammatory signaling pathways. One potential GMM that causes muscle wasting in the elderly have not been evulatued is TMAO. Background color in pink indicates cellular events and signaling pathways associated with muscle wasting, and background color in blue indicates those related to chronic heart failure. Please see the text for detail description. Please note that several signaling pathways such as MAPK, mTOR and SMAD2/3 are also involved in heart failure. However, the ligands trigger these signaling pathways leading to heart failure are different from those activate the pathways in muscle wasting. (Created with BioRender).

The signaling molecules that have been investigated include androgen, growth hormone (GH), insulin-like growth factor-1 (IGF-1), ghrelin, and myostatin, among many others [Figure 1]. Androgen, especially testosterone, is an anabolic hormone is naturally deceased with age in men. Low plasma testosterone level is associated with many metabolic syndrome and CVD^[18]^ and is correlated with exercise intolerance in men with HF^[19]^. Although the impact of low plasma testosterone levels on skeletal muscle in older adult remains controversial, administration of testosterone has been observed to enhance physical performance of HF patients, e.g., quadriceps strength, walking test and oxygen consumption in several clinical studies^[20–24]^. Studies have demonstrated that testosterone can activate multiple cell signaling pathways associated with cardiac and skeletal hypertrophy^[25,26]^. Testosterone elicits its function via interacting with intracellular androgen receptor (AR). The activation of AR then exert its transcriptional function to active genes associated hypertrophy or interact with the signaling molecules involved in mammalian target of rapamycin (mTOR) and mitogen-activated protein kinases (MAPK) pathways^[26,27]^, which are two important signaling transduction pathway in regulating muscle cell growth^[28]^. Insulin and IGF-1, are two well-known protein ligands that promote protein synthesis. They do so by activating the phosphoinositide 3-kinase-serine/threonine-protein kinase/mTOR (PI3K-AKT-mTOR) and MAPK pathways^[28]^. Activation of these pathways mediate the expression of myogenesis regulatory factors and contribute to dynamic protein balance in muscle cells^[29–31]^. Moreover, Activation of insulin/IGF-1 signaling pathways can ameliorate ubiquitin-proteasome-mediated proteiolysis as well as myostatin (MSTN) associated atrophic signaling that we will discuss below^[30,32]^. Ghrelin, an acylated peptide, stimulates the secretion of GH, cortisol, aldosterone, catecholamines, and prolactin. The release of GH can induce the secretion of IGF-1. It regulates appetite and food intake. Therefore, the reduction of the ghrelin level is linked to patients with HF and sarcopenia.

On the other hand, MSTN (also known as GDF8), belongs to the transformation growth factor β family functions via binding to the activin receptor type 2B recptor to activate actviate the mothers against decapentaplegic homolog 2 and 3 (SMAD2/3) signaling pathway and is a negative regulator of skeletal muscle growth. Overexpression of MSTN signaling in mice leads to muscle atrophy^[33]^, whereas MSTN-knockout mice develop abnormal skeletal muscle hypertrophy^[34]^. It was reported that the MSTN level is increased in the hearts and in plasma of patients with HF^[35,36]^ or in the skeletal muscle of rodent models with chronic HF^[37]^. Therefore, MSTN has been proposed as a potential therapeutic target in managing sarcopenia and HF.

### Mechanisms involved in skeletal muscle metabolism

Besides pathways that link to skeletal muscle growth and development, studies have also supported the notion that increasing protein degradation or decreasing protein synthesis and cell death are major cellular events accruing in sarcopenia [Figure 1]. Specifically, three main cellular processes associated with skeletal muscle wasting, namely the ubiquitin-protease system (UPS), the autophagy-lysosome pathway (ALP), and apoptosis, are clearly altered in sarcopenic patients with HF.

#### Ubiquitin-protease system

Ubiquitin-protease system (UPS) is a major cellular proteolytic system responsible for degrading the misfolding proteins, cell cycle regulators, and transcription factors^[38]^. In UPS, the most well-known ubiquitin enzymes associated with muscle atrophy are two E3 ubiquitin-protein ligases, TRIM63 (Tripartite Motif Containing 63, or MuRF1), and FBXO32 (F-box only protein 32, or atrogin 1). The substrates for TRIM63 are myofibrillar proteins, such as titin, myosin heavy chain and light chain, and myosin-binding protein C, whereas FBXO32 targets factors that regulate proteins synthesis. Once recognizing their protein targets, they add multiple ubiquitin to the target proteins. The polyubiquitinated protein targets are then degraded via proteasomes^[39]^. Expression of TRIM63 and FBXO32 can be regulated by NF-kB (transcription factor nuclear factor-kB) and the FOXO (forkhead box group O) transcription factor family, which can be induced by pro-inflammation cytokines such as tissue necrosis factor alpha (TNF-α), interleukin-6 (IL-6), and interleukin-1β (IL-1β). Interestingly, UPS and the pro-inflammation cytokine are also increased in the skeletal muscle of patients with HF, suggesting the common regulatory networks between HF and sarcopenia.

#### Autophagy-lysosome pathway

Another important system for protein breakdown in skeletal muscles is the ALP, which is a non-specific cellular process to degrade cytoplasmic damaged organelles and cellular molecules in the lysosome. Misregulation of autophagy in muscle causes mitochondrial damage, endoplasmic reticulum stress, impaired sarcomeric-protein turnover, and cell death^[40]^. It was reported that the system contributes to the protein degradation that occurs in denervated muscle in rodent models^[41,42]^. Physiologically, ALP is essential for muscle maintenance. Using fluorescence-activated cell sorting technique and naturally aged mice that contained green fluorescent protein on an autophagosome marker, LC3, García-Prat *et al*. demonstrated that the autophagy-lysosomal pathway prevents muscle stem cells from entering cell senescence^[43]^, which is a common cellular event present in the sarcopenic muscle at geriatric age^[44]^. Therefore, ALP may promote the stemness of stem cells allowing muscle regeneration efficiently. The function of ALP declines during aging in skeleton muscle, and the regeneration ability is also impaired in aging muscle. These studies demonstrated that ALP plays a critical role in maintaining muscle homeostasis and regeneration. However, several genetic models and human studies have shown the activation of ALP in muscle cells contributes to muscle wasting and atrophy. In addition, cathepsin L, a lysosomal protease in ALP system, was shown to be increased in catabolic muscle atrophy in rat models^[45]^. Constitutively active a transcription factor FOXO3, which leads to muscle atrophy, induces autophagy-related genes e.g., *LC3* and *Bnip3*^[46]^. Using an oxidative stressed induced-muscle atrophy transgene mouse model, superoxide dismutase protein (*SOD1^G93A^*), Dobrowolny *et al*., showed that ALP was activated in *SOD1*^*G93A*^ transgenic mice and reduced autophagy using shNRA-mediated knock-down *LC3* restored muscle mass in transgene^[47]^. LC3 is also upregulated in different muscle atrophy states, such as cachexia, diabetes, and fasting^[48]^. Autophagy can also remove organelles selectively via a specific pathway, such as mitochondria via mitophagy, which is a necessary pathway to eliminate dysfunctional mitochondria and promote the renewal of these organelles. Dysregulation of mitochondria functions is well known for causing aging-associated diseases, including sarcopenia. In conclusion, the proper function of ALP is not only required for skeletal muscle homeostasis but also plays a role in muscle atrophy under catabolic conditions.

#### Myocyte apoptosis

Another mechanism associated with muscle atrophy and sarcopenia is myocyte apoptosis. Studies have been shown that a higher frequency of apoptosis of skeletal myocytes can be found in patients with HF or in animal models compared to the control groups^[49–52]^. Several common alterations of signaling pathways that occur in HF and sarcopenia can lead to cell apoptosis, particularly in the presence of the pro-inflammation signaling pathway. Evidence has been reported that the circulation level of TNF-α, C-reactive protein (CRP), and IL-6 correlated with a functional decline of skeletal muscle and muscle mass, suggesting a direct role of these molecules in sarcopenia^[53,54]^. Additionally, circulating concentrations of TNF-α trigger myocyte apoptosis in HF^[55]^. The lower grade of inflammation creates an oxidative stress environment that produces reactive oxygen and species (ROS), triggering the muscle atrophy program by activating the FOXO transcription factor family, which were discussed earlier. Therefore ROS has been linked to exercise intolerance, impaired mitochondria function^[56]^. Several studies have revealed that many mechanisms may contribute to the alteration of muscle metabolism in patients with HF. It was shown that oxidative phosphorylation is attenuated in patients with HF, energy transfer via mitochondrial creatine kinase is impaired, and overall ATP levels are reduced^[57]^. All these phenomena observed in patients with HF and sarcopenia may be linked to mitochondrial dysfunction in myocytes. Thus, understanding mitochondrial function and regulation may help us to better understand the mechanism underlying muscle wasting in sarcopenia and HF, which we will discuss further below.

## CONTRIBUTIONS OF THE GUT MICROBIOME IN HEART FAILURE AND SARCOPENIA

The gut hypothesis of human disease can be traced back to nearly 2500 years ago when the ancient Greek physician Hippocrates claimed that “*All disease begins in the gut*” Increasing evidence supports the involvement of the gastrointestinal system in the pathogenesis of many human diseases, including HF and various aging-related conditions. First, alterations made in diet as well as aging itself can influence the composition of the gut microbiota. Second, the composition of the gut bacteria population is different between healthy and CVD subjects. Despite some well-recognized limitations, studies comparing the gut microbiota profiles in stool samples between healthy subjects and patients with CVD revealed the decreased diversity of the gut microbiota and alteration of microbiota composition^[58]^. Lastly, the gut metabolite generated by the gut microbiome can affect the host’s physiology and pathophysiology. For example, lipopolysaccharide (LPS) and trimethylamine *N*-oxide (TMAO), can induce inflammation while short-chain fatty acids (SCFAs) and bile acids (BA) can effect on host metabolism^[59]^.

### The gut-heart axis: interplay between the gut microbiome and cardiac aging and disease

Various studies over the past decade have linked gut microbiota with pathogenesis of heart disease, especially in the setting of HF^[60–62]^. Alterations of the gut microbiota can lead to the development of risk factors for atherosclerotic vascular disease and directly impact pathogenic disease progress such as acute coronary syndromes and HF^[63]^. In the gut-heart axis scenario, gut bacteria and their endotoxin products, i.e., LPS, may translocate to the host circulation due to the impairment of the gut barrier function that abnormally increased the intestinal permeability, which occurs in patients with HF. This bacterial translocation may trigger low-grade systemic inflammation, alter the immune system, and can lead to increased disease severity in patients with HF. Several animal studies and association studies in humans support this hypothesis. By comparing biomarkers profiles for patients who had chronic HF with or without recent-onset peripheral edema and healthy individuals, Niebauer *et al.* reported that the endotoxin concentration and pro-inflammatory cytokine are significantly increased in patients who had chronic HF with peripheral edema but not in other groups^[64]^. The increased endotoxin can be normalized by a short-term diuretic treatment but not for cytokine^[64]^. Recently, Beale *et al.* demonstrated that patients with HF with preserved ejection fraction had a depletion of SCFA-producing bacteria that generate SCFA, known to have a beneficial and vital role for cardiovascular homeostasis in small cohorts from two independent communities^[62]^. Several microbiome-derived metabolites have been demonstrated their effects in the heart. We will discuss them below.

#### Trimethylamine N-oxide

Among the gut microbiome-derived metabolites in human diseases, TMAO is the most notable one. TMAO is the compound generated from trimethylamine (TMA)-containing dietary nutrients such as choline, betaine, and L-carnitine. It was recently demonstrated that a microbial gene cluster, *gbu*, is responsible for converting an intermediate product of dietary carnitine, γ-butyrobetaine to TMA^[65]^. The circulating TMA is then converted into TMAO by FMO3 (hepatic flavin monooxygenase 3) in the hosts’ liver. Work from our groups and others has demonstrated that the TMA-TMAO pathway as a novel connection between diet the gut microbiota and the risks of atherosclerosis^[66,67]^ and thrombosis^[68–70]^. For example, elevation TMAO level in plasma was found to be associated with an increased risk of occurrence major adverse cardiovascular events (MACEs) in a large cohort study^[60]^. A smaller cohort study also revealed that the increased plasma level of TMAO was associated with acute coronary syndromes and can be used as a prognostic marker for MACEs^[71]^. Additionally, using an untargeted metabolomics approach, our group has identified that TMAO and N6,N6, N6-trimethyl-L-lysine (trimethyl lysine, TML), a TMAO-producing nutrient precursor, can serve as predictors for MACEs^[67,72]^. Moreover, association studies linked TMAO with an increasing level of CRP and LPS to contribute to chronic inflammation and endothelial dysfunction^[68,73]^.

How does TMAO cause cardio-metabolic diseases? Chen *et al.*, demonstrated that the pathophysiological level of TMAO can promote ER stress by directly binding to the endoplasmic reticulum stress kinase PERK (EIF2AK3) and inducing the expression level of FOXO1 in hepatocytes to cause hyperglycemia and metabolic dysfunction^[74]^. Other potential mechanisms for TMAO have been discussed, such as activation of pro-inflammation pathway causing fibrosis^[53]^, promoting microvascular dysfunction in the heart tissues of patients with HF without the presence of coronary disease, and having a synergized effect on neurohormonal derangements^[75]^. However, mechanic studies are needed to further demonstrate the direct detrimental effect of TMAO in these processes in humans. Meanwhile, TMAO has been associated to cardiovascular aging. Plasma level of TMAO was shown to be increased in the elderly, both in humans and rodent models^[76]^. The elevated circulating TMAO level was shown to contribute to endothelial dysfunction, a characteristic of the aging process, in aged rats, possibly through increasing vascular inflammation and oxidative stress^[76]^. Long-term feeding mice with TMAO induces aging-like vascular endothelial dysfunction through reduced nitro oxide (NO) bioavailability and promoted oxidative stress in young mice and healthy humans^[77]^. The studies suggested that TMAO may play a role in the development of age-related diseases.

#### Short-chain fatty acids

According to the American Heart Association diet and lifestyle recommendations that high-fiber diets have a beneficial association with a lower risk of developing CVD^[78]^. Although the beneficial effects of high-fiber diets remain to be explored, recent attention has been focused on the effect of SCFAs on the host’s physiology. As mentioned above, SCFAs are the end-products from the fermentation processes of gut microbiota and are derived from the non-digestible soluble fibers such as cellulose and other complex carbohydrates that can’t be used by the host^[79]^. High-fiber diets increase the diversity of the gut microbiome and are favored for SCFA-producing bacteria, while low-fiber diets are linked to gut dysbiosis and hypertension^[80]^. The majority of SCFA products in the gut are acetate, propionate, and butyrate. It is now clear that SCFAs have anti-inflammatory effects as well as profoundly regulate the host’s immune system. How does SCFA modulate inflammation and the host’s immune system? Strong evidence support that SCFAs act as signaling molecules binding to their well-known G-protein-coupled receptors (GPCR), GPR41, GPR43^[81]^, GPR109A^[82]^ and OLFR78^[83]^ in animal models. However, the precise mechanism remains unclear. Mechanically, it was demonstrated that SCFAs bind to their GPCR, e.g., GPR41/43, to regulate its downstream signal molecules such as activation of p38 and suppression of AKT and ERK pathways. As a result, it downregulates the expression of inflammatory cytokines such as IL-1α, IL-1β and Intercellular Adhesion Molecule 1, reduces the production of chemokine, and attenuates TNF-α associated inflammation pathways^[84,85]^. Moreover, the activation of GPR41/43 can increase the interaction of β-arrestin with I-kBα. This interaction, thus, reduces the phosphorylation of NF-kB and downregulation of IL-1β and Monocyte chemoattractant protein-1 secretion^[86,87]^. The beneficial cardiac effects of SFCAs were also documented. A study done by Marques *et al.* demonstrated that dietary fiber and acetate supplementation in deoxycorticosterone acetate (DOCA)-salt hypertension mouse model not only increases SCFA-producing bacteria in the gut but also reduces blood pressure, cardiac hypertrophy and fibrosis, and improves cardiovascular function^[88]^. Similar results were also reported in a cardiac injury rat model induced by angiotensin II (Ang II). Sodium butyrate (NaBu) feeding in an Ang II-induced cardiac injury rat model attenuated cardiac hypertrophy and suppressed fibrosis and inflammation. Interestingly, this inhibition of inflammation by NaBu may work through the cyclooxygenase (COX)/prostaglandin E2 (PEG2) pathway, which is associated with the ERK pathway^[89]^. The anti-inflammation effect of SCFAs may also act at the epigenetic level. It is known that butyrate is a potent histone deacetylase (HDAC) inhibitor. The inhibition of HDAC increases acetylation of histones that enhance the accessibility of the chromatin and thus upregulation of gene expression^[90]^. In vitro cell culture experiment showed that Nabu suppressed Ang II-induced cardiac hypertrophy by inhibiting COX/PGE2 pathway in an HDAC5/HDAC6-dependent manner^[62]^. SCFA treatment in LPS or TNF-α exposed human umbilical vein endothelial cells reduced the inflammation-associated genes, such as IL-6 and vascular cell adhesion molecule 1 as well as the HDAC activity^[91]^. It was demonstrated that butyrate elicits its anti-inflammatory effects via the inhibition of HDAC in a bone marrow-derived macrophages (BMDM) model stimulated by LPS. The recruitment of polymerase II to the promoter of the inflammation genes, e.g., IL-6, interleukin-12, and Nos2, was reduced compared to the non-butyrate treated LPS-BMDMs^[92]^. Taken together, SCFAs act in the host to modulate its metabolism and immune function and contribute to the host’s health. SCFA supplementation can improve metabolic status in HF conditions. However, more clinical studies are needed to support such intervention in humans.

#### Bile acids

Bile acid (BA), especially the secondary BA converted by the gut microbiota, is linked to HF. The primary BA, which is normally stored in the gallbladder, is synthesized in the liver from cholesterol and forms the major component of bile. The function of primary BA is to emulsify dietary fats and to facilitate the absorption of lipids and lipid-soluble vitamins. Because diet and low-density lipoprotein (LDL)-cholesterol (LDL-C) are risk factors for developing CVD, dietary strategies to reduce LDL-C levels to prevent disease are a recommendation for people with a high level of blood lipids hyperlipidemia and hypertriglyceridemia. The impact of diet and lipid metabolism of in CVD has been reviewed^[93,94]^. In this review, we focused on the gut microbiota-derived BA on CVD and aging. The secondary BAs is included deoxycholic acid (DCA), lithocholic acid (LCA), hyodeoxycholic acid, and ursodeoxycholic acid are converted by gut microbiota-derived enzymes in the colon^[95]^. Bile salt hydrolase and 7α and 7β dehydoxylase are responsible for the generation of secondary BAs in the gut^[96]^.

It is well known that secondary BAs act as signaling molecules that interact with cell-surface and nuclear hormone receptors to modulate dietary lipid metabolism^[97]^ and energy homeostasis^[98]^ and potentially affecting CVD pathogenesis. Elevated serum BAs have been long speculated to affect cardiac function. Several in vitro cell culture studies showed that the cardiomyocyte function, such as contraction action potential, was impaired upon various BAs treatment^[99–103]^. Additionally, in a BA-overload mouse model in which *Fxr* (farnesoid X receptor) and *Shp* (small heterodimer partner), two critical nuclear receptors responsible for maintaining bile acid homeostasis, are deleted, Desai *et al.* showed that the double knockout mutants (DKO) display cardiac hypertrophy, bradycardia and exercise intolerance^[104]^. When treating this DKO mouse with cholestyramine, a bile acid-binding resin known to reduce the circulating BA pool, the cardiac dysfunction phenotypes were restored, suggesting BA is directly responsible for the cardiac dysfunction presented in DKO mice^[104]^. A small clinic exploratory study reported that the ratio of secondary to primary BAs was increased in patients with chronic HF^[105]^. The ratio was associated with reducing overall survival on univariate analysis but not significant after adjustment for clinical characteristics and NT-proBNP. Whether changes in bile acids promote disease progression and sarcopenia in HF and aging beyond their metabolic effects remains unknown at this point. Clearly, more clinical studies with a large sample size are required to confirm the data as well as explore the regulation and potential role of BA metabolism in HF.

#### Dietary polyphenols

Dietary polyphenols, particularly ellagitannins (ETs), have been shown to have an antioxidant and anti-inflammatory effect that may have a beneficial role on the skeletal muscle and heart during the aging and disease process^[106,107]^. ETs can be found in pomegranates, berries, walnuts, and oak-aged red wines^[108]^. Urolithin A (UroA) and urolithin B (UroB) are two major gut metabolites produced from ETs-containing foods^[109]^. Administration of Uro A and UroB was shown to suppress the pro-inflammatory cytokine fractalkine and a sarco/endoplasmic reticulum Ca^2+^ ATPase, SERCA2, in the mouse heart as well as restore the mechanical properties and calcium dynamics of cardiomyocyte^[110]^. In a surgical induced myocardial reperfusion injury mouse model, it was demonstrated that UroA reduced ROS reaction via controlling PI3/AKT pathway and enhancing antioxidant activity^[111]^. Additionally, Ghosh *et al.* showed that UroA supplementation delayed the onset of muscular aging by increasing adenosine triphosphate (ATP) and nicotinamide adenine dinucleotide (NAD+) levels in murine skeletal muscle^[112]^. It was shown that UroA elicits its beneficial effect via enhancing mitochondria health, particularly via increasing mitochondria mitophagy. However, the mechanical insight of how UroA improves mitochondria health in cardiac aging, heart disease and sarcopenia remains to be explored.

#### Phenylacetylglutamine

Other gut microbial metabolites (GMM) associated with CVD have recently been reported. Using untargeted metabolomics, Nemet *et al.* reported that the plasma metabolite Phenylacetylglutamine (PAGln) was associated with CVD and major adverse cardiac events, which confirmed prior observations in patients with chronic kidney diseases where PA Gln was considered a uremic toxin^[113]^. The microbial gene, *porA*, is responsible for covert dietary phenylalanine to PAGln. It was shown that PAGln accelerated platelet clot formation and enhanced thrombosis potential in mice, likely via through its homology to adrenergic receptor ligands to the α2A, α2B, and β2 adrenergic receptors^[113]^. Mechanistically, how PAGln can drive disease progression in CVD remains an area of active investigation.

#### Other protein-bound uremic toxin

Two of most investigated protein-bound uremic toxin (PBUTs), indoxyl sulfate (IS) and p-cresyl sulfate (pCS), have been shown to predict clinic outcomes in patients with chronic kidney and heart diseases and correlate with cardiovascular events and all-cause mortality^[114–116]^. IS is derived from bacterial metabolism of dietary tryptophan to indole and pCS is synthesized by intestinal bacteria from tyrosine and phenylalanine. Both substances are normally cleared by renal proximal tubular secretion but accumulate in the circulation once renal function is impaired^[117]^. Both substances also have been demonstrated to have cardiac adverse effect. The profibrotic prohypertrophic and pro-inflammatory effect of IS was first demonstrated by Krum group more than decade ago in vitro cell culture system. Treatment of cardiac cells stimulate cardiac fibroblast collagen synthesis, hypertrophy and activate the gene expression level for key inflammatory cytokine such as TNF-α, IL-6, and IL-1β^[118]^. Animal study using Dahl salt-resistant normotensive IS-administered rats also demonstrated that IS aggravates cardiac fibrosis and hypertrophy and enhance the oxidative stress^[119]^. The parent from of PCS, p-cresol, was shown to activate of Ca^2+^-dependent protein kinase Cα that lead to reduction of the contraction rates and disassembling of connexin 43 gap in the culture cardiomyocytes^[120]^. Moreover, it was shown that PCS promote cardiac apoptosis, affected function of the left ventricle of the heart in the mice that underwent 5/6 nephrectomy^[121]^. PBUTs play a role in vascular dysfunction^[122]^. However, their effect on cardiac aging have not been evaluated. Because PBUTs also promote cardiac inflammation or induce oxidative process, which are two major cellular events occurred in aging process, one would assume these compounds may play a role in cardiac aging. Interestingly, it was recently reported that Klotho, an anti-aging protein with reno/cardio-protective effects, can suppress the IS-induced inflammatory response to protect against renal fibrosis and cardiac hypertrophy by promoting M2 macrophage polarization^[123]^. Interestingly, Klotho deficiency was recently shown to cause cardiac aging via affecting the molecules involved in the against oxidative stress^[124]^. The restoring Kloth function might be an alternative intervention for organ dysfunction assorted with aging.

### The gut-muscle axis: interplay between gut microbial metabolism and muscle wasting

Emerging evidence in animal models has shown that gut microbiota-derived metabolites have a direct effect on muscle wasting. Siddharth *et al.*, found that aged rat with sarcopenia displayed a distinct gut microbiome compared to rats with a normal muscle mass^[125]^. The muscle dysfunction is correlated with the downregulation of genes involved in the vitamin, carbohydrate, protein, and lipid biosynthesis and metabolism, which may contribute to the regulation of muscle mass. In an acute leukemia-induced muscle wasting mouse model (BAF3), feeding mice with *Lactobacillus* species, which were reduced in the BAF3 mice versus the control mice, reduced the inflammation and attenuated muscle atrophy^[126]^. In another study, treating mice with *Faecalibacterium prausnitzii* reduced adipose tissue inflammation, increased gastrocnemius muscle mass, and the expression of mitochondrial respiratory chain protein in high-fat-fed mice^[127]^. The administration of *Lactobacillus reuteri* to a cancer cachexia mouse model, which exhibited adipose tissue and muscle atrophy phenotypes, can increase muscle mass and fiber size^[128]^. Feeding naturally aged mice with butyrate, a gut microbiota-derived SCFAs and HDAC inhibitor, increased muscle fiber cross-sectional area and prevented intramuscular fat accumulation in the old mice^[129]^. We have discussed the effect of SCFA above. In a genetic knockout mouse model in which ghrelin, a hormonal peptide functions as a GH stimulator, was deleted, it was shown that the old ghrelin KO mice developed microbial dysbiosis and worsened muscle atrophy compared to the former wildtype mice under fast-induced conditions^[130]^ All these studies highlight the role of gut microbiota-derived metabolites in regulating skeletal muscle metabolism.

Inflammation signaling pathway is one of the key factors of the imbalance in skeletal muscle metabolism leading to muscle wasting. Therefore, GMM that can modulate inflammation signaling pathways, such as SCFAs, can affect normal skeletal muscle metabolism and cause muscle wasting. Indeed, various studies have been demonstrated that SCFAs play a profound role in regulating muscle mass and physical function^[131]^. There are studies that suggest that liver disease, which causes abnormal production of BAs, may lead to muscle wasting and cachexia^[132,133]^. However, currently, there is no study evaluating the GMM-BAs associated with sarcopenia. Although the role of TMAO in CVD and other metabolic diseases is studied extensively, the link between TMAO and muscle wasting in sarcopenia remains unclear. An association study recently reported that the plasma level of TMAO was significantly and independently increased in frail patients with CVD than in non-frail ones^[134]^. However, since the study did not collect other important data for evaluating the relationship between dietary and muscle wasting, such as nutritional data and body composition of patients, further studies with such information are required to study the role of TMAO in sarcopenia.

Interestingly, gut microbiome-derived uremic toxins, IS, were shown to be associated with sarcopenia^[135,136]^. By treating C2C12 myoblast cells with six-protein-bound uremic toxins, Enoki *et al.* identified that IS may act a potent uremic toxin that promotes developing skeletal muscle atrophy^[135]^. It was shown that IS inhibited myoblast proliferation as well as myotube differentiation and formation by inducing oxidative stress-mediated expression of myostatin and atrogin-1 and inflammatory cytokine expression IL-6 and TNF-α in muscle cells^[135]^. Moreover, chronic administration of IS increased myostatin and atrogin-1 expression levels and decreased muscle mass in mice. Similar results were also reported by another study, and it was shown that cell apoptosis was increased in IS-treated myoblasts^[136]^. Additionally, Sato *et al.* demonstrated that IS induced metabolic alteration in C2C12 myotubes by upregulating glycolysis, upregulated glycolysis, pentose phosphate pathway and glutathione metabolism and decreased energy generation pathways^[137]^. Studies suggest that IS may serve as a key pathogenic factor for sarcopenia. Understanding the mechanism by which the IS-induced muscle atrophy can open up the potential therapeutic strategies for treating muscle wasting and sarcopenia.

Several clinical studies also revealed the importance of GMM in muscle wasting. A recent study done by Picca *et al.* showed that old people with physical frailty and sarcopenia have a different abundance of the gut microbiome, suggesting the changes in the gut microbial composition is associated with sarcopenia^[138]^. In a randomized, double-blind trial study, in which the efficacy of prebiotic Darmocare Pre® was evaluated in sixty older individuals aged 65 and older. Although the prebiotic did not significantly improve frailty in order adults, the score for exhaustion and handgrip strength was improved^[139]^. However, in a three-month intake of synbiotic supplementation study, the body composition and inflammatory status did not change in the elderly but showed an improvement of hydration status. Additionally, Aoyagi *et al.* reported that drinking fermented milk with different frequencies does not alter muscle mass in the elderly^[140]^. It seems that there is a discrepancy among the clinical trial studies. However, all these human studies were performed on a variety of populations and conditions with different measurement criteria. Therefore, a large cohort multicenter study with systematic evaluation methods should be performed in the future to confirm the beneficial effect of probiotics on improving muscle function and physiology in sarcopenia.

## GUT MICROBIAL METABOLITES AND MITOCHONDRIAL FUNCTION IN CARDIAC AGING AND MUSCLE WASTING

Both heart and skeletal muscle are high-energy demand organs. They use ATP as the energy for myocyte contraction to allow skeletal muscle movement and locomotion and the heart to pump the blood to the whole body. Each heartbeat costs about 300 mg of ATP in humans, while the demand of ATP increases more than 100 times in skeletal muscle during intensive exercise^[141,142]^. Mitochondria are double-membrane organelles and the energy production center of the cells. They also play a vital role in many cellular processes, including cellular redox homeostasis, ion homeostasis, calcium storage and homeostasis and regulation of cell death and survival. Mitochondria produce ATP via oxidative phosphorylation (OXPHOS), which takes place in the inner mitochondrial membrane. During OXPHOS, electrons derived from NADH and FADH2 combine with molecular oxygen by entering the electron transport chain. During this process, protons are pumped to the intermembrane space of mitochondria that produce a membrane potential serving as energy to the synthesis of ATP from ADP. OXPHOS also generate byproduct reactive oxygen species (ROS), which are known to regulate many physiological functions in normal and disease process. Mitochondria also serve as sensors and regulators of calcium signaling, which is an essential mechanism to maintain intracellular calcium homeostasis as well as regulate energy production^[142,143]^.

Mitochondrial dysfunction has been considered as an underlying cause of myocyte loss during cardiac aging, failure, and sarcopenia^[144,145]^. Studies from animal and human studies link mitochondrial energetics and control of muscle mass and cardiac aging. The Baltimore Longitudinal Study of Aging demonstrated that mitochondrial function declines with age and may contribute to age-associated loss of muscle function and cardiorespiratory fitness^[146]^. Using unbiased transcriptomic and targeted quantitative metabolomics profiling, Lai *et al.*, showed that mitochondrial energy metabolic derangements, such as the lower amount of many Kerbs cycle intermediates and elevation in lactate and acylcarnitine species, occur during the early development of HF induced by pressured overload mouse model, suggesting a bottleneck of carbon flux into mitochondria^[147]^.

Mitochondria dynamics, which involves mitochondria fusion and fission, is important for mitochondrial maintenance. The imbalance of the fusion and fission process is a feature for cell senescence. Evidence from mouse genetic models in which genes encoded mitochondrial fusion or fission proteins were mutated also demonstrated the importance of mitochondria during aging and sarcopenia. For example, the ablation of OPA1 mitochondrial dynamin like GTPase induced FOXO signaling and unfolded protein response which caused catabolic program of muscle loss and systemic aging^[148]^ and mice without mitochondrial pro-fusion factors such as *Mfn2* (mitofusin 2) in skeletal muscle developed significant defects of muscle growth and sarcopenia^[149]^. The impact of mitochondrial dynamics in aging and sarcopenia was reviewed^[150,151]^.

In aging or stressed myocytes, the elevation of ROS levels, increasing of oxidative stress and overload of Ca^2+^ uptake are present in mitochondria. The elevation of these cellular events causes the opening of mitochondrial permeability transition pore (mPTP) and thus loss of the mitochondrial membrane potential. The high ROS level and loss of the mitochondria membrane potential can cause reduction of ATP production, trigger mitophagy, cell death and impaired mitochondrial biogenesis. These cellular events can affect muscle metabolism leading to muscle atrophy, cardiac aging and failure. For example, excessive mitochondrial ROS trigger protein degradation systems, ALP and UPS^[152,153]^ and reduced ATP production can activate the AMP kinase -FOXO3 pathways leading to upregulation of protein degradation systems^[46,154]^ that were known for affecting muscle metabolism as we discussed earlier.

In dysfunctional mitochondria, the microconidia contents, such as mitochondria genomics, ATP, and transcription factors, may be released to the cytosol to trigger various pro-inflammatory signaling. These leaking microdontia contents serve as damage-associated molecular patterns (DAMP) to activate nucleotide-binding oligomerization domain (NOD)-like receptor protein 3 (NLRP3) associated inflammasomes intracellular multiprotein complexes and enhance the inflammatory response of the cells^[155]^.

Alteration of mitochondrial biogenesis is another feature of age-related mitochondrial dysfunction. Mitochondrial biogenesis generates new mitochondria through multiple transcriptional regulatory pathways, the transcription of nuclear genes for biogenesis, import of nuclear-encoded mitochondria proteins and transcription and replication of mtDNA^[156]^. PGC-1α (peroxisome proliferator-activated receptor-gamma coactivator α) is the master regulator in mitochondrial biogenesis. PGC-1α activates the transcription and transcriptional activity of the nuclear respiratory factor-1 and -2 (Nrf-1 and Nrf-2) and cooperates with NRF1/2 and related receptor alpha (ERRα) to activate mitochondrial biogenesis cascade. NRFs then activate the mitochondrial transcription factor A (Tfam) to activate the mitochondrial genome by triggering replication and transcription of the corresponding regions of mtDNA^[157]^. Evidence from human and animal studies also revealed that mitochondria biogenesis plays a role in pathophysiologic processes in sarcopenia and cardiac aging. The aging heart displays a reduced level of PGC-1α along with its downstream targets involved in mitochondria biogenesis^[158–160]^. The decline of mitochondria biogenesis was observed in the aging skeletal muscle and heart. In a multi-ethnic study of human sarcopenia versus age-matched controls in muscle biopsies, the transcriptome profiles of genes involved in mitochondria biogenesis, e.g., PGC-1α and ERRα nuclear receptor, were reduced in sarcopenic individuals^[161]^. Another study by Kong *et al.* also observed the key biogenesis regulator, PGC-1α, and its downstream effector and a major mitochondrial deacetylase, SIRT3^[162]^, were dramatically reduced in the muscle fibers taken from elderly participants compared to young participants^[163]^. Similar findings were also found in a spontaneous senescence-accelerated mouse (SAM) prone 8 (SAMP8) aging mouse model, whereas the expression of PGC-1α, *Nrf-1* and *Tfam* were significantly decreased^[164]^. Taken together, proper regulation of mitochondrial function and physiology, including mitochondria dynamic, mitophagy and biogenesis, is important in healthy cardiac and skeletal muscle aging. Modulating the pathways associated with these processes may effectively improve the aging phenotype in the heart and skeletal muscle.

Studies on GMM and mitochondrial function in myocytes have been reported. It was shown that germ-free mice have a lower mtDNA content, reduced mitochondrial biogenesis markers, and oxidative phosphorylation complexes gene expression in skeletal myocytes, indicating alteration of mitochondria function and metabolism in skeletal muscle of germ-free mice^[165]^. Munukka *et al.* gave SCFA-producing bacteria *Faecalibacterium prausnitzii* to mice that were fed with a high-fat diet (HFD) and resulted in upregulation of the expression of mitochondrial respiratory chain complex and increased gastrocnemius muscle mass^[127]^. Administration of UroA in mice fed with HFD improves skeletal muscle function in aged mice compared to that in the control HFD-fed mice only^[166]^. However, treating mice with TMAO impaired b-oxidation in cardiac mitochondria, deceased OXPHOS, and pyruvate metabolism^[167]^. Cardiomyocytes treated with a pathological concentration of chenodeoxycholic acid, a bile acid, suppressed the expression of genes involved in fatty acid oxidation in mitochondria^[104]^. GMM-derived uremic toxin, IS, was shown to cause mitochondrial dysfunction and ATP shortage in muscle cells^[137]^. These preclinical studies demonstrated that GMM have a profound impact on mitochondrial function in skeletal muscle and hearts.

## POTENTIAL THERAPEUTIC STRATEGIES FOR IMPROVING GUT DYSBIOSIS IN AGING POPULATION

It is clear that aging is linked to gut dysbiosis^[168,169]^. As discussed above, several GMMs can induce inflammation, which is associated with multiple diseases in the elderly. Chronic low level of inflammation is a hallmark of human aging. Therefore, strategies that can reduce the production of GMM-associated inflammation or promote bacteria that can attenuate inflammation may hold promising ways to improve the overall physiology performance of old adults. Here we list two potential gut microbiome-based therapeutic strategies for the aging population and discuss their limitations.

### Physical exercise strategy

Aging is associated with reduced muscle function and atrophy. The correlation between physical exercise and improvement of muscle size and overall physical function is well established. Evidence also supports the notion that physical exercise is beneficial for improving aerobic capacity, metabolic regulation cardiovascular function, and muscle mass and function^[170–173]^. Additionally, several studies have shown that there is a strong association between exercise and a health-beneficial gut microbiome, suggesting physical exercise increase the growth of beneficial gut microbe^[174–177]^. However, such relationships remain associative in nature and more human studies are required to confirm if the beneficial effects of physical exercise on physical performance and cardiac function are modulated by gut microbial metabolism.

### Nutritional strategy

Because the production of GMM is highly related to what we eat daily and some GMM is associated with CVD and aging-associated diseases, a nutrition-based approach maybe one of the most efficient ways to modify the gut microbiome and GMM. It is well established that high-fiber diets favor SCFA-producing gut microbe, and may promote an anti-inflammation effect (see Section The gut-heart axis: interplay between the gut microbiome and cardiac aging and disease). Thus, high-fiber diets are becoming an important strategy to improve health conditions in our daily life, particularly for the elderly. While several animal studies have demonstrated a high-fiber diet can increase muscle fiber and improve physical function, it remains unclear if the beneficial effects of high-fiber diets on increasing muscle mass and function are directly mediated by gut microbial metabolism.

Another nutritional strategy is calorie restriction (CR). CR is a primary dietary intervention shown to lower the risk of CVD and increase the healthy life span^[178]^. Animal studies indicate that CR can promote the growth of several health-beneficial microbes^[179–182]^. For example, Fraumene *et al.* have demonstrated that CR fosters the growth of Lactobacillus in a rat study^[183]^. Although animal and human trial studies suggest that CR may be a feasible strategy to improve the physical performance of older adults, more research remains needed to determine the suitably restricted calories for each older individual to avoid deteriorating their physical condition.

Lastly, oral intake of probiotics, prebiotics, or synbiotics have been considered as an effective and non-invasive option for countering aging pathways. Theoretically, this strategy can increase the populations of health-beneficial gut microbes such as *bifidobacteria* or *lactobacilli* in older adults^[184–186]^. However, the positive effect is more significant on mental and digesting system health^[185–188]^. For example, Inoue *et al.*, conducted a randomize, double-blind placebo control trial by providing Probiotic *bifidobacteria* supplementation or placebo to 38 elderly for 12 weeks, and they found that probiotics supplement combined with moderate resistance exercise may improve the mental condition and bowel movement^[188]^. In contrast, the protective effect against sarcopenia and HF from consuming probiotics, sytiotics or prebiotics remains inconclusive, and the presence of an intact gut microbiome may deter any substantial changes in gut microbial populations.

## CONCLUSION

The gut-muscle and gut-heart axis play key roles pathophysiology of aging-associated heart disease and sarcopenia. While there is ample evidence showing the correlation between GMM and aging-associated heart and muscle diseases, precisely how they interact to promote sarcopenia remains poorly understood. More mechanical and *in vivo* studies are necessary to dissect the underlying mechanisms of how GMM regulates myocyte (in the heart and skeletal muscle) during aging and pathophysiological processes at cellular and molecular levels. Nevertheless, there is tremendous promise for dietary or therapeutic interventions to target gut microbiome-associated metabolic pathways with GMM as mechanism biomarkers that can identify underlying pathophysiologic processes that link sarcopenia with HF and aging.

## Data Availability

Not applicable.
